# Exploring the Relationship Between the *How to Eat* Intervention and Eating Competence Among Repeat Dieters

**DOI:** 10.3390/nu18030368

**Published:** 2026-01-23

**Authors:** Cristen Harris, Ellyn Satter

**Affiliations:** 1 Food Systems, Nutrition, and Health Program and Department of Epidemiology, School of Public Health, University of Washington, Seattle, WA 98195, USA; 2 Ellyn Satter Institute, Madison, WI 53711, USA; ellyn@ellynsatterinstitute.org

**Keywords:** eating competence, eating attitudes, eating behaviors, restrictive eating, dieting, weight-neutral, size-inclusive, disordered eating, employee wellness

## Abstract

**Background/Objectives**: The 10-session *How to Eat* intervention was developed to institute Eating Competence (EatC) and repair distorted eating attitudes and behaviors growing out of chronically restrained eating and/or repeated weight reduction dieting. *How to Eat* was conducted over a 12-year period as an employee wellness option at two locations in the midwestern United States. **Methods**: Participants in *How to Eat* were adult employees of their respective hospital or university who voluntarily enrolled after screening and assessment by each site facilitator. Pre- and post-measures were the 16-item EatC measure, the 26-item Eating Attitudes Test (EAT-26), and body weight. **Results**: In the hospital setting, a total of 43 adults participated, with a mean (±*SD*) age of 47.5 ± 10.7 years, primarily female (95.3%) and white (90.7%). *How to Eat* was associated with a significant increase in EatC total scores (22.8 ± 6.5 to 34.3 ± 4.9) and a decrease in EAT-26 scores (10.7 ± 8.1 to 3.7 ± 2.9), both *p* < 0.001. In the university setting, a total of 52 adults participated, 89.4% female, with a mean (±*SD*) age of 39.3 ± 11.4 years. University participants were significantly younger, *p* < 0.001. *How to Eat* was also associated with a significant increase in EatC total scores (24.1 ± 7.0 to 36.6 ± 6.9) and EAT-26 scores (13.9 ± 8.8 to 3.2 ± 4.2), both *p* < 0.001. At both sites, changes in total EatC, Contextual skills, and EAT-26 scores had strong effect sizes. Mean body weight was ±5% pre/post-intervention at either site. **Conclusions**: *How to Eat* is associated with clinically significant improvements in measures of EatC and a decrease in eating disturbances among repeat dieters without significantly impacting body weight. Positive results from employee wellness settings support future experimental studies with more diverse samples and additional outcome measures.

## 1. Introduction

A high proportion of adults make repeated attempts at weight loss through chronically restrained eating and/or weight reduction dieting [1]. Some if not many of these repeat dieters present clinically with distorted eating attitudes and behaviors [2,3]: They can no longer diet and do not know how to eat. They feel conflicted, negative, guilt-ridden, and ambivalent about eating [4,5,6]. They show a typical pattern of eating restraint and disinhibition as they go on and off diets and lose and regain body weight [7,8]. They have lost their fine tuning with food regulation and can only respond to extremes of hunger and fullness [9]. They function adequately, socially and emotionally, so are not eating-disordered. Yet, recurrent, self-perceived “failures” by repeat dieters may harm mental and/or physical health over the long term [8,10,11,12,13]. Those who are amenable to treatment are willing to set aside weight loss as an outcome goal and address their distorted eating attitudes and behaviors.

*How to Eat* supersedes conflict and anxiety about eating with the positive eating attitudes and behaviors consistent with Eating Competence (EatC), described by the Satter Eating Competence Model (ecSatter) [14]. *How to Eat* is a weight-neutral strategy, focusing on overall health and well-being independent of body size. Weight-neutral strategies reduce internalized weight stigma and have positive psychological effects that lead to improved eating behaviors, body acceptance, self-esteem, wellness, and mental health [12,15]. Weight-neural approaches are also associated with lower morbidity [16,17] and lower mortality risk [18,19], regardless of body size.

EatC is predicated on trust in the utility and effectiveness of biopsychosocial processes to guide what and how much to eat and what to weigh. Those processes are hunger and the drive to survive, appetite and the need for pleasure, the social reward of sharing valued food, and the biological propensity to maintain a stable body weight range [14]. EatC has four domains: (1) positive attitudes about food, eating, and eating enjoyment; (2) food acceptance attitudes and skills that support eating an increasing variety of available food; (3) the skills and resources needed for managing the food context and organizing reliable eating times; and (4) internal regulatory attitudes and skills that facilitate being aware of and responding to body cues as a guide to consuming enough enjoyable food to satisfy hunger and appetite [14].

Eating-competent adults were more likely than non-eating-competent to engage in a variety of health-supportive behaviors. They had higher dietary quality [20], better food resource management skills, were less likely to consider themselves as being food-insecure [21,22], and ate more fruits and/or vegetables [21,22,23]. They showed greater evidence of social and emotional health [21,22,24], exhibited fewer eating disorder symptoms [21,22,24], were more likely to engage in physical activity [23,25,26], had greater body satisfaction [21,22,24,27], and had better sleep quality and quantity [25,28]. Eating-competent adults had the same or lower BMIs [21,22,28], lower lipids and fasting blood glucose levels [29], fewer diabetes symptoms [28,29], lower blood pressures [30], better oral health [31], and greater executive function skills [32], which may play a role in managing food context. In short, eating-competent adults consistently exhibited beneficial physical, nutritional, biochemical, and mental health characteristics and organizational skills.

*How to Eat* is an experiential intervention developed by Ellyn Satter (2nd Author) to target the distorted eating attitudes and behaviors of repeat weight loss dieters. *How to Eat* uses evidence-based cognitive and behavioral intervention to institute EatC: cultivate positive attitudes about eating, establish internally directed eating, and support dependable access to rewarding food. The intervention encourages structured availability of preferred food coupled with strong permission to eat what and as much as desired at consistent times. To clarify: The goals of *How to Eat* are not to get participants to eat certain amounts or types of food or to weigh within certain limits.

Evaluation is an integral component of the *How to Eat* intervention. This article begins a reporting process with the intent of laying the groundwork for future controlled studies using *How to Eat.* For this report, *How to Eat* was offered to employees at two different locations over a 12-year period (2007–2019), in a hospital system and a public university, and by two different trained facilitators. For both facilitators, who were registered dietitians (RDs), a minority of their employee-patients qualified for the *How to Eat* intervention. As a consequence, it took several years to accumulate the volume of unpublished observational evidence reported in this article. Results are described separately for each location.

## 2. Materials and Methods

### 2.1. Study Characterization and Ethical Aspects

This is a retrospective evaluation of the single-arm *How to Eat* intervention using two non-probabilistic convenience samples from clinical practice to explore the relationship between participation in *How to Eat* and changes in EatC and in distorted eating attitudes and behaviors. Participants were U.S. adults who were repeat dieters; were employees of either a hospital system or a public university where *How to Eat* was offered; and voluntarily engaged with their respective employee wellness programming. This study utilized only the de-identified sets of employee wellness data that were collected over a 12-year period in accordance with ethical standards. Participants voluntarily enrolled and consented to the intervention, but consent was not required for this evaluation, because of the anonymity of the de-identified data sets utilized. The Human Subjects Division of the first author’s (C.H.) institution determined that this evaluation did not involve human subjects, as defined by U.S. federal and Washington state regulations, and that full board review and approval by the IRB was not required (Study No. 00024575). Evaluation of the university-derived data was additionally approved by that university’s IRB (Study No. 00005047). Permission to use the instrument to measure EatC was obtained by each intervention facilitator.

### 2.2. Screening, Assessment, and Enrollment

Adults who were candidates for *How to Eat* were screened for inclusion criteria, assessed for fit with a weight-neutral intervention, and invited to participate. Persuasion or recruitment was strictly avoided. Repeat dieters typically are unaware of the negative impact of long-term repeat dieting on their eating attitudes and behaviors and weight stability. The dream of weight loss is powerful, and that awareness did not necessarily allow them to give up dieting and pursue a weight-neutral option.

Hospital employees were referred to the RD/intervention facilitator by their primary care practitioners for weight management. University employees self-referred through word-of-mouth, health fairs, university newsletters, email announcements, and workplace flyers. In both settings, the RDs conducted a brief in-office or phone screening to determine if inclusion criteria were met: adult employees (18 years or older) who reported repeat dieting, expressed conflict and anxiety about eating, were English-speaking, and were not pregnant. If these criteria were met, then potential participants were invited to be assessed for *How to Eat*.

Assessment examined long-term eating and weight-related attitudes and behaviors with an individual interview and questionnaires. The *How to Eat* packet, which was completed at home, included a questionnaire collecting basic demographic information (age, gender, race/ethnicity, employee status); detailed questions on experiences with weight loss dieting and weight history; a measure of EatC [21,22,33,34]; and a screening inventory for disturbed eating patterns [35]. If the assessment indicated distortion in eating attitudes and behaviors that did not meet criteria for a clinical eating disorder [36], the RD offered the *How to Eat* intervention. Those who chose to enroll expressed a willingness to work toward the goal of making eating and physical activity positive and consistent without striving for weight loss; and were committed to attending all intervention sessions.

### 2.3. Measurement Tools

Eating Competence. The original EatC measure, ecSI [21,22,33], and the revised measure, ecSI 2.0^TM^ [34], are 16-item instruments with demonstrated retest reliability [37] and construct validity in free-living adults [21,22,33,34]. Since the revised measure was not available until 2019 [34], data for this study were collected using ecSI [21,22,33]. Sample items include Contextual skills, “I have regular meals;” Eating attitude, “I am comfortable about eating enough;” Food acceptance, “I experiment with new food and learn to like it;” and Internal regulation: “I eat as much as I am hungry for.” For each item, participants selected from 5 response options scored on a 4-point Likert scale: 0 (never), 0 (rarely), 1 (sometimes), 2 (often), or 3 (always). Total scores range from 0 to 48 with a score of 32 or greater identified as being eating-competent. Four subscales comprise the dimensions of EatC, but the subscales have not been normed. The factor re-analysis published in 2019 resulted in moving one item from the Internal regulation subscale to the Eating attitudes subscale [34], but the raw data were not available to re-calculate subscale scores for this study. Therefore, only the Contextual skills (0 to 15) and Food acceptance (0 to 9) subscales are reported.

Disturbed Eating Patterns. The Eating Attitudes Test, EAT-26, was used to screen out individuals with eating disorders. This is a 26-item instrument with established reliability and validity [35]. Sample items include, “Am preoccupied with a desire to be thinner,” “Find myself preoccupied with food,” and “Feel extremely guilty after eating.” Participants select from 6 response options for each item, scored on a 4-point Likert scale: 0 (never), 0 (rarely), 0 (sometimes), 1 (often), 2 (usually), or 3 (always). Total scores range from 0 to 78 with higher scores indicating more severely disturbed eating patterns. The survey evaluates disturbed eating patterns in 3 main areas: (1) dieting, or the avoidance of fattening foods and preoccupation of being thinner; (2) bulimia and food preoccupation, reflecting thoughts of food and bulimia; and (3) oral control, or the self-control of eating and the perceived pressure from others to gain weight. Total scores greater than 31 indicate “extreme disturbance, very likely disordered eating,” 21–30 is “significant disturbance, likely disordered eating,” 11–20 is “moderate or normative disturbance,” and 0–10 is “normal, modest to low anxiety” with eating [35].

Body Weight. Body weight was measured and recorded at baseline, weekly, and upon completion of *How to Eat* to assess weight stability in the context of permission to eat. Weighing was conducted in privacy by each facilitator using a balance-beam scale that was calibrated quarterly. Participants were asked to remove outerwear (e.g., coats and jackets) and shoes prior to measurements. Weight was recorded in pounds to the nearest quarter of a pound.

### 2.4. Intervention

*How to Eat* is an experiential intervention to establish EatC: positive and intentional eating as conceptualized according to ecSatter [14]. It addresses specific deficits common among repeat dieters: (1) impaired ability to internally regulate food intake based on hunger, appetite, and satisfaction; (2) inconsistency with providing rewarding food in ample amounts; and (3) conflict and anxiety about eating, manifested as self-imposed lack of permission to eat certain foods (e.g., high-calorie “forbidden” or “treat” foods) accompanied by feeling out-of-control around such foods.

*How to Eat* is grounded in cognitive/behavioral techniques [38,39,40] that are systematically and progressively applied to target conflict and anxiety about eating at the same time as it sequentially builds EatC. While such techniques are frequently used by mental health professionals, *How to Eat* does not attempt to change individuals’ underlying emotional functioning and/or life circumstances. As a consequence, *How to Eat* is accessible for health professionals not trained in mental health intervention. Participants are supported in feeding themselves reliably by being given strong permission to eat personally preferred food and encouraged to have sit-down meals and snacks. *How to Eat* processes include systematic desensitization (progressive relaxation paired with repeated, gradual exposure to anxiety-provoking food), self-awareness training (mindfulness and feedback), and self-acceptance training (setting aside judgments). Other techniques include positive reframing (giving reasonable explanations for negative behavior), and shaping eating (building EatC in small, successive steps). Experiential tools for change include (1) in-session focused eating exercises (FEEs), applying the processes just described, using food that is increasingly challenging to the participant (i.e., self-identified “out-of-control,” “forbidden,” or “treat” foods); (2) in-session discussions tightly focused on task achievement and eating-related behavior, cognitions, and emotions; (3) progressive take-home assignments focused on task achievement; and (4) education reinforcing insights and behavior change.

*How to Eat* was facilitated by two RDs who were trained in a master class [41]. Individual *How to Eat* sessions lasted ~50 min for a total of 10 sessions. Each session reviewed task achievement assigned in the previous session and explored feelings, thoughts, and experiences associated with implementing that task. The core activity for each session was an FEE in which participants were systematically desensitized to their negative thoughts, feelings, and memories related to food and eating and supported in self-awareness and self-acceptance. During the FEE, participants were guided by the facilitator in relaxation training, repeated neutral exposure to food, and increased their self-awareness about eating and themselves. Food exposure started at baseline with neutral, relatively unchallenging food (e.g., crackers) and, in subsequent weeks, gradually expanded towards what each participant deemed increasingly challenging food. Insights about eating were gained through a process of discovery and supported with *How to Eat* handouts offered at the end of the session. Each session concluded by establishing individualized eating and physical activity tasks for the following week.

At the end of the intervention, participants again completed the EatC and EAT-26 questionnaires and had body weight measured by the facilitator.

### 2.5. Statistical Analyses

Data were summarized as means and standard deviations for continuous variables or as frequencies and proportions for categorical variables. Descriptive statistics were used to evaluate differences in participant characteristics, *t*-tests for continuous variables, and chi-square tests for categorical variables. Continuous variables (EatC scores, EAT-26 scores, and body weight) were tested for normality, skewness, and kurtosis. Relationships between continuous variables were explored using Pearson’s *r* correlation, and those between groups using independent-samples *t* tests. Paired, two-tailed *t* tests were used to evaluate differences in pre- and post-intervention measures. Effect size was calculated using Cohen’s *d*, and interpreted as a small (0.20), medium (0.50), or large (0.80) effect [42]. Given the small sample sizes, all tests were confirmed using the nonparametric Wilcoxon signed-rank test. To assess the temporal stability of the intervention effects, we examined the association between year of intervention completion and pre/post-difference scores for each outcome variable. Scatterplots with fitted linear trends were used for visual inspection of outcomes, accompanied by linear regression models with completion year as a predictor. Data was analyzed with the IBM Corp Statistical Package for Social Sciences version 28.0.0 (IBM SPSS Statistics, Chicago, IL, USA). An alpha level of 0.05 was used for all statistical tests.

## 3. Results

### 3.1. Hospital System Results

Participants who completed all 10 sessions of the *How to Eat* intervention (*n* = 43) from 2007 to 2013 had a mean age of 47.5 years old (*SD* 10.7, *range* 26 to 65), were primarily female (95.3%), and were white (90.7%). Group comparisons were not made, because of the small number of non-female (*n* = 2) and non-white (*n* = 3) participants. The completion rate was 89.6%, with 5 people dropping out between orientation and completion. Reasons for dropping out included a change in job status or schedule, loss of interest, and the inability to set aside weight loss as a goal. Data was unavailable for non-completers; therefore, results include only those who completed 10 sessions.

Pre- and post-intervention, 9.3% (*n* = 4) versus 100% (*n* = 43) of participants had EatC scores of 32 or higher and were, therefore, identified as eating-competent. Pre-intervention scores ranged between 23 and 37; post-intervention scores ranged from 33 to 45. Table 1 shows that when compared to baseline, post-intervention EatC scores averaged significantly higher and with large effect sizes for EatC total scores (*t*(42) = 12.20, *p* < 0.001, *d* = 1.9) and both of the reported subscales, Contextual skills (*t*(42) = 8.56, *p* < 0.001, *d* = 1.4) and Food acceptance (*t*(42) = 4.34, *p* < 0.001, *d* = 0.7).

Post-intervention EAT-26 scores were significantly lower than baseline scores (*t*(42) = 6.53, *p* < 0.001, *d* = 1.0), also with strong effect. To examine the distribution of eating-related disturbances more closely, Table 2 shows the relative frequencies of EAT-26 eating disturbance levels. Pre-intervention EAT-26 scores indicated that 41.9% of participants had “moderate” to “significant” disturbances related to eating with scores ranging from 11 to 30. By the end of the intervention, 93.0% had “normal, modest to low anxiety,” (scores 0 to 10) and only 7.0% had “moderate” disturbance with eating (scores 11 to 20). Results of Pearson correlations revealed weak and non-significant relationships between EatC and EAT-26 scores at both pre-intervention (*r*(41) = −0.081, *p* = 0.125) and post-intervention (*r*(41) = −0.049, *p* = 0.398).

Complete body weight data were available for only half of the participants (*n* = 22) due to data loss prior to de-identified data sharing. Of those retained, measured body weight was similar (*t*(21) = 0.314, *p* = 0.757, *d* = 0.1) between pre- and post-intervention. Upon examining the available weight data individually, each participant maintained their weight within ±5% of their initial, pre-intervention body weight. Data for EatC scores, weight, and pre-intervention EAT scores were minimally skewed (−1 to +1) or kurtotic (−1 to +1); only the post-intervention EAT-26 scores were moderately skewed and kurtotic. Similar results were obtained with Wilcoxon signed-rank tests. Therefore, means and standard deviations are reported to facilitate comparisons with published EatC literature.

### 3.2. Public University Results

A total of 52 employees participated in the *How to Eat* intervention at the public university from 2008 through 2019. Participants had a mean age of 38.3 years old (*SD*, 11.4; *range*, 21 to 62), were primarily female (86.5%), and completed an average of 9.3 sessions (*SD,* 3.2; *range,* 3 to 18). An independent-samples *t*-test indicated that the participants from the university were significantly younger than those enrolled through the hospital system (*t*(93) = 3.999, *p* < 0.001). Group comparisons were not made, because of the small number (*n* = 7) of non-female participants.

Pre- and post intervention, 19.2% (*n* = 10) versus 84.6% (*n* = 44) of participants had EatC scores of 32 or higher and were, therefore, identified as eating-competent. Pre-intervention scores ranged between 8 and 36; post-intervention scores ranged from 19 to 48. Table 1 shows that when compared to baseline, post-intervention EatC scores averaged significantly higher for EatC total scores (*t*(51) = 13.77, *p* < 0.001, *d* = 1.9) and both of the reported subscales, Contextual skills (*t*(51) = 7.61, *p* < 0.001, *d* = 1.0) and Food acceptance (*t*(51) = 3.80, *p* < 0.001, *d* = 0.5). Effect sizes were strong for EatC total and Contextual skills and medium for Food acceptance.

Table 1 shows that the *How to Eat* intervention at the university was associated with overall improvements in EAT-26 scores with large effect size (*t*(51) = 8.78, *p* < 0.001, *d* = 1.2). Table 2 shows that the beginning and ending proportion of those identified as having “moderate” to “extreme” disturbances in eating behaviors dropped from 61.6% to 5.8%. Results of Pearson correlations of EatC and EAT-26 scores revealed a weak and non-significant relationship pre-intervention (*r*(50) = −0.206, *p* = 0.775), but the post-intervention correlation was moderate, inverse, and significant (*r*(50) = −0.473, *p* < 0.001).

Body weight was similar (*t*(51) = 1.19, *p* = 0.241, *d* = 0.2) between pre- and post-intervention. Examination of the individual weight data shows that each participant, except for one, maintained their weight within ±5% of their initial, pre-intervention body weight. The one participant who lost 11% of their body weight from pre- to post-intervention was measured at 530 lbs. at baseline. Data for EatC scores and pre-intervention EAT scores were minimally skewed (−1 to +1) or kurtotic (−1 to +1); the post-intervention EAT-26 scores and pre- and post-weight scores (due to 2 outliers) were moderately skewed and kurtotic. Therefore, results were confirmed with Wilcoxon signed-rank tests, and means and standard deviations are reported.

### 3.3. Temporal Trends

**Figure 1:**
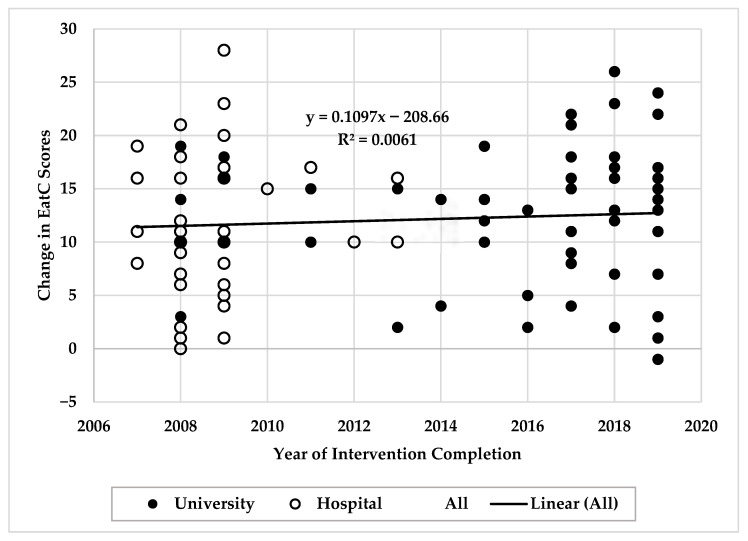
Scatterplot of pre/post-change in EatC total scores by year of *How to Eat* intervention completion in a hospital or university setting. Each point represents one participant. Solid line indicates fitted linear regression trend. No systematic temporal trends were observed across change scores.

Visual inspection of pre/post-change scores plotted against year of intervention completion revealed no systematic temporal trends in EatC total scores (see Figure 1), Food acceptance, Contextual skills, or body weight. Consistent with this, linear regression analyses indicated that year of completion of the *How to Eat* intervention was not a significant predictor of the change in EatC total scores (*β* = 0.110, *SE* = 0.146, *p* = 0.453), Food acceptance (*β* = −0.056, *SE* = 0.045, *p* = 0.214), Contextual skills (*β* = −0.049, *SE* = 0.076, *p* = 0.519), or body weight (*β* = 0.346, *SE* = 0.259, *p* = 0.186). However, linear regression analysis did indicate that year of intervention was a significant predictor of change in EAT-26 scores (*β* = −0.532, *SE* = 0.180, *p* = 0.004). See Supplemental Materials for scatterplots with fitted trendlines for Food acceptance (Figure S1), Contextual skills (Figure S2), body weight (Figure S3), and EAT-26 (Figure S4) scores across years of intervention completion.

## 4. Discussion

The primary aim of this retrospective evaluation of the *How to Eat* intervention was to explore the relationship between participation and changes in eating attitudes and behaviors. The clinical data analyzed was from the *How to Eat* individual intervention offered repeatedly by two trained RDs [41] in their respective practices over a 12-year period. The results of this evaluation suggest that the intervention was unusually successful at increasing EatC and reducing disturbed eating.

*How to Eat* was offered to adults with a history of repeated dieting and distorted eating attitudes and behaviors as part of employee wellness programming in a hospital system and a public university setting. The effect sizes were large, suggesting that this is a clinically important intervention. By the end of the *How to Eat* intervention, most participants reported exceptional improvements in eating attitudes and behaviors as well as considerable moderation in the disturbed eating patterns commensurate with eating disorders. These positive reports were accompanied by no significant differences in body weight measured at the start and end of the intervention. This was despite the intervention’s strong permission to eat what and as much as desired at regular eating times.

*How to Eat* was correlated with considerably greater changes than those of other interventions. Average EatC total scores at both sites increased by ~12 points and 93% of participants’ EAT-26 scores decreased to the lowest level of disturbance, “normal, modest to low anxiety.” In contrast, educational interventions with EatC showed average increases of at most 4 points [43,44,45,46]. Cognitive behavioral interventions with bulimia show typical EAT-26 improvement to the second-lowest level of “moderate or normative disturbance” [47].

*How to Eat’s* impressive comparable results likely grew out of the experiential nature of the intervention as well as cooperation with biopsychosocial drives. Internally regulating food intake, trusting appetite, and letting weight be what it will be entail cooperating with the body’s homeostatic mechanisms rather than trying to ignore and overwhelm those processes. To that end, the experienced RDs who conducted *How to Eat* were consistent in giving strong permission to eat, systematically extinguishing restrained eating, and sanctioning participant weight. This is in contrast to other interventions that contained prescriptive elements contradicting trust in biopsychosocial drives. Educational interventions included stipulations of what and/or how much to eat and tended to be didactic rather than experiential [43,44,45,46,48]. Cognitive–behavioral interventions were predicated on following a “healthy eating pattern,” decreasing binge eating, or achieving certain weight outcomes [47].

The intervention-related changes were generally stable across most outcomes examined, suggesting consistency of effects across the 12-year study period. One outcome, change in EAT-26 scores, demonstrated a significant association with year of intervention completion, indicating potential temporal variability. EAT-26 scores may be more sensitive to subtle changes in measurement context, participant characteristics, or respondent interpretation over time compared to the other outcomes. This possibility is supported by a recent study on the psychometric properties of the EAT-26 in which the authors highlighted concerns in using this measure when assessing people with BMI-defined “overweight” or “obesity” seeking weight loss treatment [49], similar to the participants in this study. Although recent evidence demonstrates that EAT-26 scores are consistent across time [50], the temporal variation observed in this study may, instead, reflect broader contextual shifts that differentially influenced this measure. Since the magnitude of the observed trend was small, and given the small sample size and exploratory nature of these analyses, this finding must be interpreted with caution. The stability of the remaining outcomes—particularly the EatC total scores and its two reported subscales—supports the overall strength of the intervention effects.

EatC correlates with nutritional, medical, and psychosocial benefits without deliberately working toward a specific dietary pattern, weight, and/or other wellness-related outcomes. In fact, working directly toward those outcomes is contradictory to the model [14]. In addition, the children of eating-competent adults have lower nutritional risk than those with fewer EatC-related capabilities [51]. Eating-competent adults perform better with parenting and feeding in that they are more likely to have family meals, create a positive feeding environment, serve and eat vegetables, and follow the Satter Division of Responsibility in Feeding [52,53], which may ultimately influence children developing EatC [54]. Therefore, facilitating EatC in employees who are also parents may have mutigenerational value and benefits.

Focusing on overall health and wellbeing independent of weight loss is the treatment of choice for repeat dieters [12]. *How to Eat* demonstrated considerable utility in that regard. Weight-neutral interventions, in general, have been shown to reduce distorted eating [55], including binge eating [15] among other beneficial outcomes. The 14-week, “Mind, Body, Food” intervention with 24 “overweight” women reported significant improvements in the ability to eat in accordance with internal regulatory cues and decreases in binge eating despite no significant change in BMI from pre- to post-intervention [56]. In a study of 38 female chronic dieters, a size-inclusive intervention demonstrated significant improvements in eating, mood, and medical parameters that were sustained at the 2-year follow-up, compared to the restrictive dieting group that sustained few improvements [57]. The size-inclusive group also maintained baseline body weight at the 6-month follow-up, while the dieting group lost weight during treatment and re-gained weight by follow-up [57]. These interventions, including *How to Eat*, provide growing evidence that weight loss is not a requisite for improved health and/or wellbeing.

Pressure on weight reduction dieting will not go away any time soon. Failing that, primary care practitioners can help neutralize harm [58]. They can identify patients who are repeat dieters, refrain from recommending weight loss, predict that still another weight loss attempt is unlikely to be “successful,” and raise the possibility of weight-neutral intervention. This retrospective evaluation gives evidence that *How to Eat* is a trustworthy resource for that weight-neutral intervention. *How to Eat* can help repeat dieters to reap the biopsychosocial rewards of becoming eating-competent. Given this encouraging evidence, experimental studies are warranted to test the *How to Eat* intervention. Such studies would include additional physical, biochemical, and mental health parameters among a varied population of adults.

### Study Limitations and Strengths

This study has a number of limitations. First, it is a retrospective analysis of an intervention that was delivered years prior to free-living adults. Causal claims are not possible, because this study is a single arm, without a control condition. The analyses relied primarily on self-reported measures, and two different versions of the EatC measure were used, which could have weakened conclusions. No follow-up data was available to determine if the associations between *How to Eat* and EatC were durable. Despite intensive training in the *How to Eat* intervention, it was delivered by two different facilitators, which may have led to some variability in implementation of *How to Eat*. The number of participants in both locations was small, overwhelmingly white and female, and living in midwestern metropolitan areas, so the results cannot be generalized to more diverse samples or in other geographical areas.

Given the length of time that data was collected, the findings may reflect changing norms, attitudes, and behaviors at different time points rather than a stable intervention effect, further limiting external validity. Loss of weight data for nearly half of the participants in the hospital system-derived dataset may have biased the body weight results, and no biochemical measures were included. Earlier participants could have influenced later participants through word-of-mouth, and those who participated may have differed systematically from those who did not, limiting internal validity. Finally, since this was a naturally occurring, small sample without a priori sample size determination, the statistical power and effect size estimates are substantially limited.

This is the first report to address the feasibility of using *How to Eat* as part of employee wellness and health promotion. One strength is that the *How to Eat* intervention is grounded in the validated ecSatter model. In addition, it was offered in naturally occurring samples of participants and facilitated by a single practitioner at each location specifically trained in *How to Eat* [41], which helped ensure quality control in the delivery of the intervention. The consistency of results across sites, the temporal stability of EatC total scores and its two reported subscales, and the large effect sizes support further investigation with randomized controlled trials applied to diverse populations and additional health-related parameters.

## 5. Conclusions

The *How to Eat* intervention, offered as a weight-neutral option for employee wellness over a 12-year period at two different locations, correlated with significant gains in EatC and resolution of eating disturbances. Findings suggest that *How to Eat* is a clinically dependable intervention associated with improved EatC and less distorted eating attitudes and behaviors in adults who are repeat dieters.

## Figures and Tables

**Table 1:** Pre- and post-intervention EatC scores, EAT-26 scores, and body weight for participants enrolled in *How to Eat* as part of hospital- and university-based employee wellness programming.

Measure	Pre-Intervention *Mean* (*SD*)	Post-Intervention *Mean* (*SD*)	Change *Mean* (95% *CI*)	*p* Value ^1^	Cohen’s *d*
Hospital (*n* = 43)					
EatC Total ^2^	22.8 (6.5)	34.3 (4.9)	11.6 (9.6, 13.5)	2.15 × 10^−15^	1.9
Food acceptance ^2^	4.2 (1.8)	5.5 (1.7)	1.3 (0.7, 1.9)	1.01 × 10^−4^	0.7
Contextual skills ^2^	5.9 (2.8)	10.1 (2.1)	4.1 (3.2, 5.1)	2.14 × 10^−10^	1.4
EAT-26 ^3^	10.7 (8.1)	3.7 (2.9)	−6.9 (−4.8, −9.1)	6.88 × 10^−8^	1.0
Weight (lbs.) ^4^	210.0 (35.1)	209.6 (34.4)	−0.4 (−2.4, 3.3)	0.757	0.1
University *(n* = 52)					
EatC Total ^2^	24.1 (7.0)	36.6 (6.9)	12.4 (10.6, 14.2)	8.23 × 10^−19^	1.9
Food acceptance ^2^	5.4 (2.1)	6.4 (1.7)	1.0 (0.5, 1.6)	3.89 × 10^−4^	0.5
Contextual skills ^2^	7.0 (3.0)	10.6 (2.7)	3.6 (2.6, 4.5)	6.05 × 10^−10^	1.0
EAT-26 ^3^	13.9 (8.8)	3.2 (4.2)	−10.7 (−8.25, −13.1)	9.04 × 10^−12^	1.2
Weight (lbs.) ^4^	212.7 (71.1)	210.9 (64.7)	−1.7 (−1.2, 4.7)	0.241	0.2

^1^ Obtained using paired-sample *t*-tests. ^2^ Total score on the 16-item measure of Eating Competence, EatC, ranges 0 to 48, and higher scores indicate more positive eating attitudes and behaviors; ≥32 is “eating-competent.” Possible scores for the 2 reported subscales are Food acceptance (0 to 9) and Contextual skills (0 to 15). ^3^ Total score on the 26-item Eating Attitudes Test, EAT-26, ranges from 0 to 78, and higher scores indicate greater disturbances with eating behaviors. ^4^ Weight was measured in pounds to the nearest ¼ pound on a balance bean scale by the facilitator; complete body weight data were available for only a subset of participants (*n* = 22).

**Table 2:** Pre- and post-intervention levels of eating disturbance for participants enrolled in *How to Eat* as part of hospital- and university-based employee wellness programming.

EAT-26 Scores ^1^ (Eating Disturbance Level)	Pre-Intervention % (*n*)	Post-Intervention % (*n*)
Hospital (*n* = 43)		
0–10 (normal)	58.1 (25)	93.0 (40)
11–20 (moderate)	27.9 (12)	7.0 (3)
21–30 (significant)	14.0 (6)	0
>30 (extreme)	0	0
University (*n* = 52)		
0–10 (normal)	38.5 (20)	94.2 (49)
11–20 (moderate)	42.3 (22)	5.8 (3)
21–30 (significant)	13.5 (7)	0
>30 (extreme)	5.8 (3)	0

^1^ Total score on the 26-item Eating Attitudes Test, EAT-26, ranges from 0 to 78, with the highest scores indicating “significant to extreme disturbance, likely to very likely disordered eating”.

## Data Availability

The aggregated data supporting the conclusions of this article will be made available by the authors upon request.

## Supplementary Materials

The following supporting information can be downloaded at https://www.mdpi.com/article/10.3390/nu18030368/s1, Figure S1: Scatterplot of pre/post-changes in Food acceptance scores by year of *How to Eat* intervention completion in a hospital or university setting; Figure S2: Scatterplot of pre/post-changes in Contextual skills scores by year of *How to Eat* intervention completion in a hospital or university setting; Figure S3: Scatterplot of pre/post-changes in body weight by year of *How to Eat* intervention completion; in a hospital or university setting; Figure S4: Scatterplot of pre/post-changes in EAT-26 scores by year of *How to Eat* intervention completion in a hospital or university setting.

## Authorization

Use of the ecSI 2.0^TM^ is free of charge, but permission to use it must be obtained from the Nutrition Education Engineering and Design (NEEDS) Center, College of Health Sciences and Technology, Wegmans School of Health and Nutrition, Rochester Institute of Technology (RIT): https://www.needscenter.org/resources/satter-eating-competence-model-ecsatter/using-ecsi-2-0/. (accessed on 20 January 2026).

## Abbreviations

The following abbreviations are used in this manuscript:

BMI	Body Mass Index
EAT	Eating Attitudes Test
EatC	Eating Competence
ecSatter	Satter Eating Competence Model
ecSI/ecSI 2.0^TM^	Satter Eating Competence Inventory
FEE	Focused Eating Exercise
