# Supplementary material for: Exploring the Relationship Between the How to Eat Intervention and Eating Competence Among Repeat Dieters
Source: Nutrients. 2026 Jan 23;18(3):368. doi: 10.3390/nu18030368 (PMC12899033; doi:10.3390/nu18030368)
Supplement: Supplementary file 1 [file nutrients-18-00368-s001.zip › nutrients-4104228-supplementary.pdf]

nutrients

MDPI

Supplementary Materials

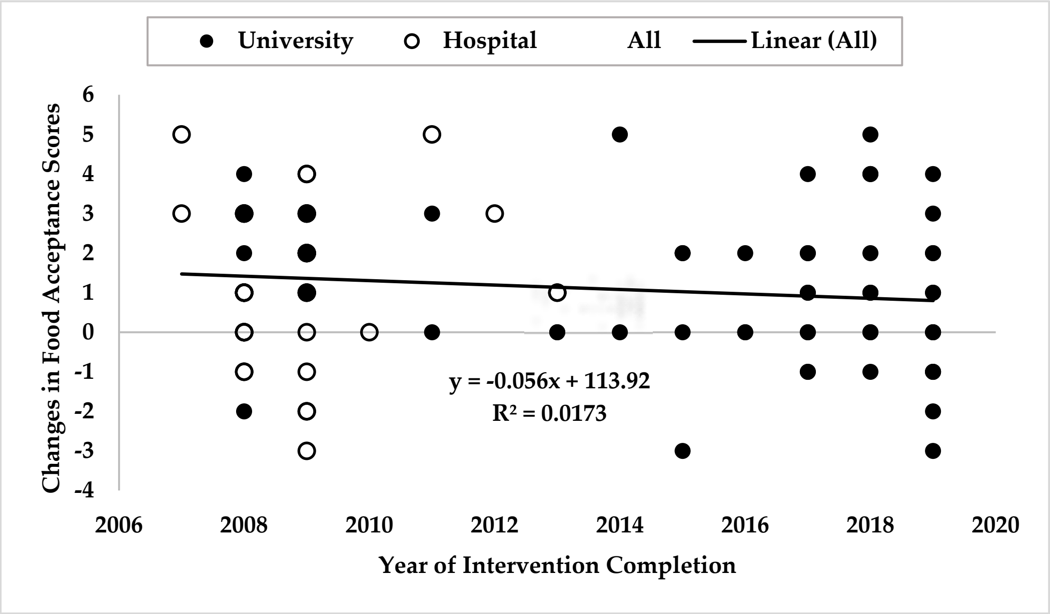

Figure S1. Scatterplot of pre-post change in Food Acceptance scores by year of *How to Eat* intervention completion in a hospital or university setting. Each point represents one participant. Solid line indicates fitted linear regression trend. No systematic temporal trend observed across change scores.

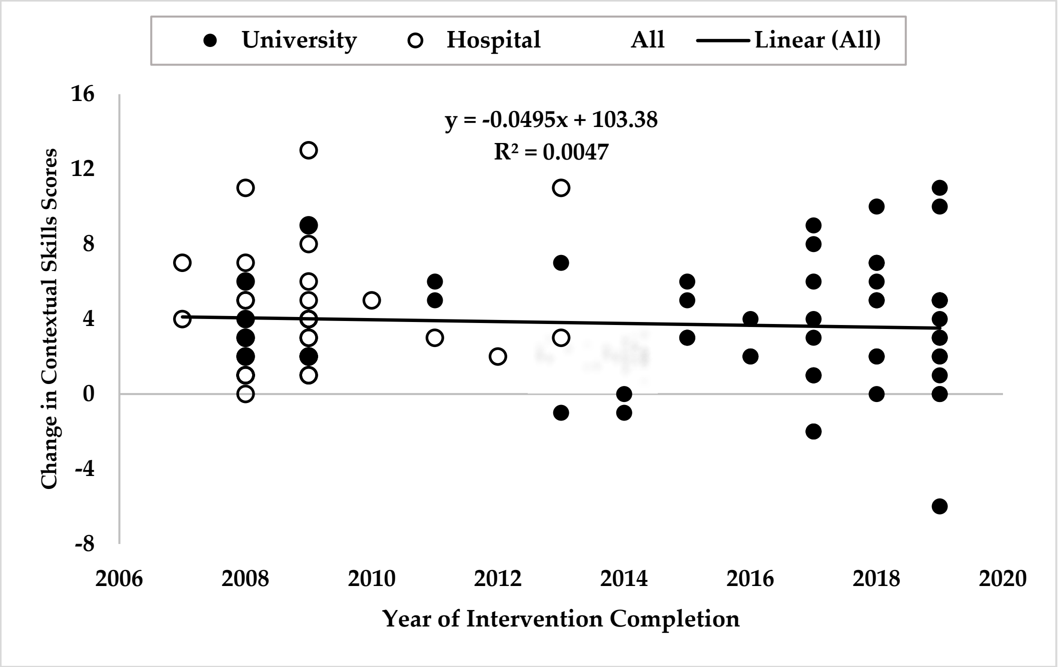

Figure S2. Scatterplot of pre-post change in Contextual Skills scores by year of *How to Eat* intervention completion in a hospital or university setting. Each point represents one participant. Solid line indicates fitted linear regression trend. No systematic temporal trend observed across change scores.

https://doi.org/10.3390/nu18030368

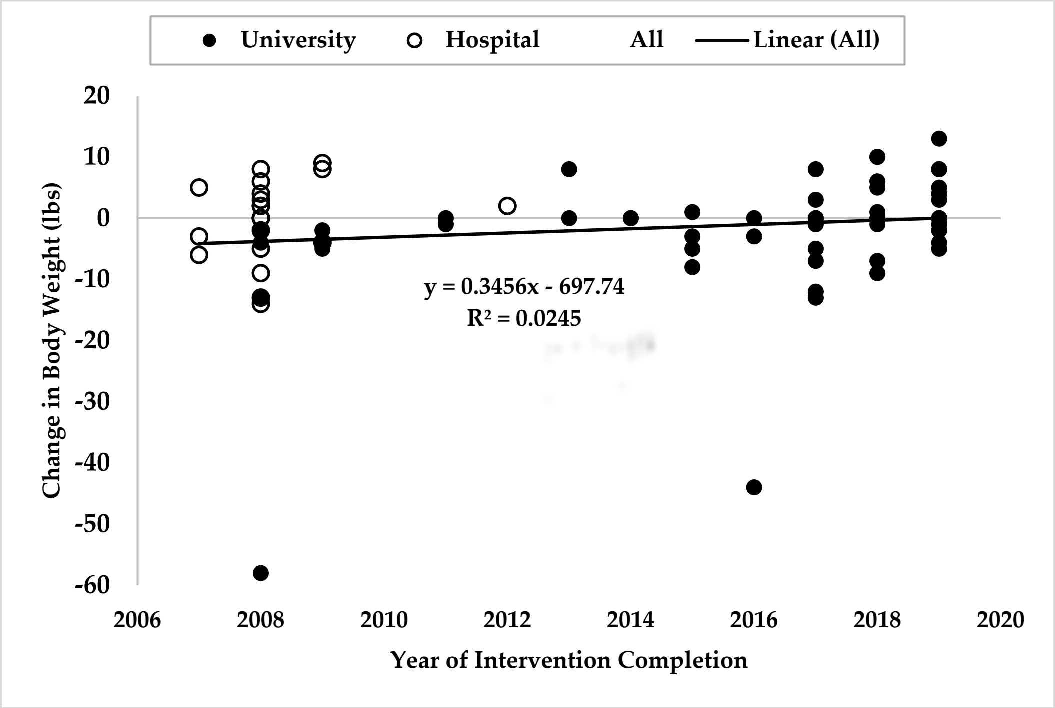

Figure S3. Scatterplot of pre-post change in body weight by year of *How to Eat* intervention completion in a hospital or university setting. Each point represents one participant. Solid line indicates fitted linear regression trend. No systematic temporal trend was observed across change scores.

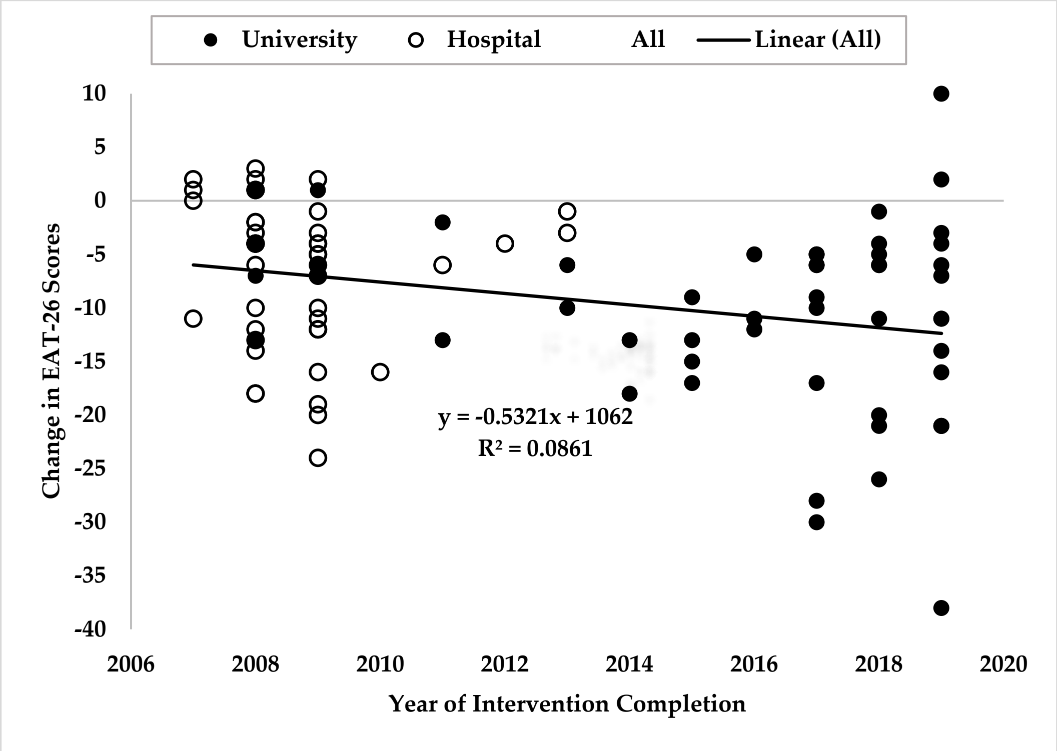

Figure S4. Scatterplot of pre-post change in EAT-26 scores by year of *How to Eat* intervention completion in a hospital or university setting. Each point represents one participant. Solid line indicates fitted linear regression trend. Systematic temporal trend was observed across change scores, $p = 0.004$.
